# Bias in Spontaneous Reporting of Adverse Drug Reactions in Japan

**DOI:** 10.1371/journal.pone.0126413

**Published:** 2015-05-01

**Authors:** Shinichi Matsuda, Kotonari Aoki, Takuya Kawamata, Tetsuji Kimotsuki, Takumi Kobayashi, Hiroshi Kuriki, Terumi Nakayama, Seigo Okugawa, Yoshihiko Sugimura, Minami Tomita, Yoichiro Takahashi

**Affiliations:** 1 Drug Safety Division, Chugai Pharmaceutical Co. Ltd., Tokyo, Japan; 2 Clinical Development Division, Chugai Pharmaceutical Co. Ltd., Tokyo, Japan; Osaka University Graduate School of Medicine, JAPAN

## Abstract

**Background:**

Attitudes of healthcare professionals regarding spontaneous reporting of adverse drug reactions (ADRs) in Japan are not well known, and Japan’s unique system of surveillance, called early post-marketing phase vigilance (EPPV), may affect these reporting attitudes. Our objectives were to describe potential effects of EPPV and to test whether ADR seriousness, prominence, and frequency are related to changes in reporting over time.

**Methods:**

A manufacturer’s database of spontaneous ADR reports was used to extract data from individual case safety reports for 5 drugs subject to EPPV. The trend of reporting and the time lag between ADR onset and reporting to the manufacturer were examined. The following indices for ADRs occurring with each drug were calculated and analyzed to assess reporting trends: Serious:Non-serious ratio, High prominence:Low prominence ratio, and High frequency:Low frequency ratio.

**Results:**

For all 5 drugs, the time lag between ADR onset and reporting to the manufacturer was shorter in the EPPV period than in the post-EPPV period. All drugs showed higher Serious:Non-serious ratios in the post-EPPV period. No specific patterns were observed for the High prominence:Low prominence ratio. The High frequency:Low frequency ratio for peginterferon alpha-2a and sevelamer hydrochloride decreased steadily throughout the study period.

**Conclusions:**

Healthcare professionals may be more likely to report serious ADRs than to report non-serious ADRs, but the effect of event prominence on reporting trends is still unclear. Factors associated with ADR reporting attitude in Japan might be different from those in other countries because of EPPV and the involvement of medical representatives in the spontaneous reporting process. Pharmacovigilance specialists should therefore be cautious when comparing data between different time periods or different countries. Further studies are needed to elucidate the underlying mechanism of spontaneous ADR reporting in Japan.

## Background

Spontaneous reporting of adverse drug reactions (ADRs) is an important data source for pharmacovigilance activities worldwide. Although spontaneous reports are useful for detecting uncommon or unexpected ADRs that occur after marketing [1], the presence of reporting bias limits the use of such reports in further analyses such as estimating ADR incidence. Previous studies have shown the various types of reporting biases, such as underreporting [2], the Weber effect [3], a tendency for serious events to be reported more frequently than non-serious events [4,5], and notoriety bias [6,7]. Ghosh and Dewanji recently showed that reporting bias can affect the results of two well-known signal detection methodologies, the Bayesian confidence propagation neural network [8] and empirical Bayes geometric mean [9,10]. Understanding the factors that affect ADR reporting is important to improve interpretation of data.

Studies in several countries have revealed factors related to a healthcare professional’s decision of whether to report an ADR [11–16]. These studies are mainly based on questionnaires given to the healthcare professionals, and few studies have used spontaneous reporting databases to confirm whether these potential factors actually affect ADR reporting [5]. Thus, the nature of reporting is still largely unknown, particularly in Japan where such studies are few. Japan also has a unique system of post-marketing surveillance, called early post-marketing phase vigilance (EPPV), which has been a condition for approval of most new molecular entities since 1 October 2001. In EPPV, medical representatives (MRs) regularly visit medical institutions during the first 6 months of marketing to collect adverse events and ADRs [17], and a positive association between the EPPV period and the number of ADRs reported has been suggested [18]. Considering that EPPV may stimulate spontaneous reporting, we expected to see the following effects of EPPV: (1) The number of ADR reports reaches its peak earlier and (2) The time lag between event onset and reporting to the manufacturer shortens.

Our hypothesis was that ADR seriousness, prominence, and frequency have the following effects on spontaneous ADR reporting attitudes:

- Serious events are more likely to be reported than non-serious events.
- Prominent (important or well known) events are less likely to be reported than uncommon events.
- Frequently occurring events are less likely to be reported than less frequent events.

Our objectives were to examine how EPPV affects ADR reporting in Japan, to describe the pattern of changes over time in post-market reporting, and to test our hypothesis that ADR seriousness, prominence, and frequency affect the rate of reporting.

## Materials and Methods

We compiled data for analysis from an ADR reporting database maintained by Chugai Pharmaceutical Co., Ltd. that includes all spontaneous reports on the manufacturer’s authorized products. We used data on ADRs from the first 2 years after marketing (study period), which enabled us to disregard potential factors, such as expanding indications, that could complicate effects on reporting and distort the results. ADRs in the database were coded according to the Medical Dictionary for Regulatory Activities (MedDRA, version 17.0) [19].

Our analysis focused on voluntary reports of events occurring with 5 drugs (capecitabine, eldecalcitol, epoetin beta pegol, peginterferon alfa-2a, and sevelamer hydrochloride) selected because they were (1) subject to EPPV, (2) not subject to all-case surveillance (because such surveillance yields only solicited reports [17]), and (3) marketed at least 6 months after the end of EPPV. Individual case safety reports compiled from these spontaneous reports were included in the analysis. Reports from the medical literature and post-marketing studies were excluded because they are not suitable for determining the characteristics of voluntary reporting.

We defined the following explanatory variable categories to classify each ADR: seriousness (serious or non-serious), prominence (high or low), and frequency (high or low). Reviewers (TN, MT, T Kawamata, SO, YS) independently classified each event as described below. Any discrepancies were resolved by discussion (details are shown in S1 and S2 Tables).

### Seriousness

The seriousness of events was evaluated according to the reporter’s seriousness assessment, which was recorded when collecting each case report. If the reporter’s assessment was unknown, we substituted the manufacturer’s assessment. ADRs were considered serious when they resulted in death; were life threatening; required hospitalization or prolongation of existing hospitalization; resulted in persistent or significant disability or incapacity; were congenital abnormalities or birth defects; or were any other medically significant events.

### Prominence

Prominence of events was determined by reviewing the following sections of package inserts issued at initial marketing approval in Japan for each product: Warnings, Important precautions, and Clinically Significant Adverse Reactions. An event’s prominence was classified as low if the event appeared in none of these sections, and it was classified as high if the event appeared in at least one of these sections.

While some labeled events refer to a single medical term, others refer to broader concepts and are listed as comprehensive events including multiple terms. For example, if the Clinically Significant Adverse Reactions section lists “liver disorder”, healthcare professionals using the package insert could envision any number of liver-related events, including hepatic failure, abnormal liver function test results, or alanine aminotransferase abnormality. Standardized MedDRA Queries (SMQs) were used as necessary to group and evaluate related terms derived from the various labeled events. SMQs comprise broad and narrow terms, but because of their higher specificity, narrow terms were used to identify the appropriate SMQs.

### Frequency

Frequency of events was determined by reviewing the frequency data included in the Clinically Significant Adverse Reactions and Other Adverse Reactions sections of each product’s package insert. ADRs listed in the highest frequency category of a frequency table were classified in the study as having high frequency. Events not in this category were classified as having low frequency. As with the prominence classification, SMQs were used as necessary to group and evaluate related events.

The number of reported ADRs was counted for each product, and the degree of change over time was examined using the following indices:

### Reporting proportion

$$\text{Reporting proportion for serious events}\left(\text{\%}\right)=\frac{\text{the number of serious events}}{\text{the total number of all events}}$$

### Serious:Non-serious ratio (odds for serious events)

$$\text{Serious}:\text{Non}-\text{serious ratio}=\frac{\text{reporting proportion for serious events}}{\text{reporting proportion for non}-\text{serious events}}$$

The High prominence:Low prominence ratio and the High frequency:Low frequency ratio were calculated similarly to the Serious:Non-serious ratio described above.

JMP software version 11.1.1 (SAS institute, Cary, North Carolina, USA) was used for the analysis.

### Effects of EPPV

We divided the study period into 3-month intervals and calculated the number of ADR reports in each interval. In a typical Weber effect pattern, the number of ADR reports peaks 1 to 2 years after marketing [3]. Thus, we evaluated whether there was a peak in reporting during the study period and, if yes, whether the peak was seen during or after the EPPV period. Because of the nature of spontaneous reporting, we were unable to include denominator values (i.e., number of patients exposed to each drug) in the analysis. As an alternative, we have described changes in the shipping volumes for each drug using the total amount of bulk powder shipped in each period. These data were extracted from periodic safety reports submitted to the Japanese health authority.

For each ADR, we calculated the time from onset to reporting to the manufacturer and considered when, in relation to EPPV, the event was reported. We calculated and assessed the proportion of ADRs reported to the manufacturer within 2 weeks of onset during the EPPV period (0–6 months) and during the post-EPPV period (7–12 months) for each drug studied. We focused on the 2-week time frame because MRs visit healthcare professionals every 2 to 4 weeks during EPPV [17].

### Ethics statement

This study protocol was approved by the institutional review board of the Public Health Research Foundation (http://www.phrf.jp/) on 1 October 2014. The board waived informed consent because the database used in this study is de-identified and includes no personal information.

## Results

Drug characteristics are shown in Table 1. The total number of ADRs included in the study was 5,967. There was a general increasing trend in shipping volumes after marketing depicted in S1 Fig.

**Table 1:** The 5 Drugs Studied.

Drug name	Date of approval	EPPV start date (= date of marketing)	EPPV end date	Total ADRs studied (n)
Capecitabine	16 Apr 2003	24 Jun 2003	23 Dec 2003	814
Eldecalcitol	21 Jan 2011	11 Apr 2011	31 Oct 2011	1,881
Epoetin beta pegol	22 Apr 2011	20 Jul 2011	31 Jan 2012	580
Peginterferon alfa-2a	16 Oct 2003	12 Dec 2003	11 Jun 2004	1,900
Sevelamer hydrochloride	31 Jan 2003	26 Jun 2003	25 Dec 2003	792

*ADR: adverse drug reaction, EPPV: early post-marketing phase vigilance.

### The effects of EPPV

**Fig 1:**
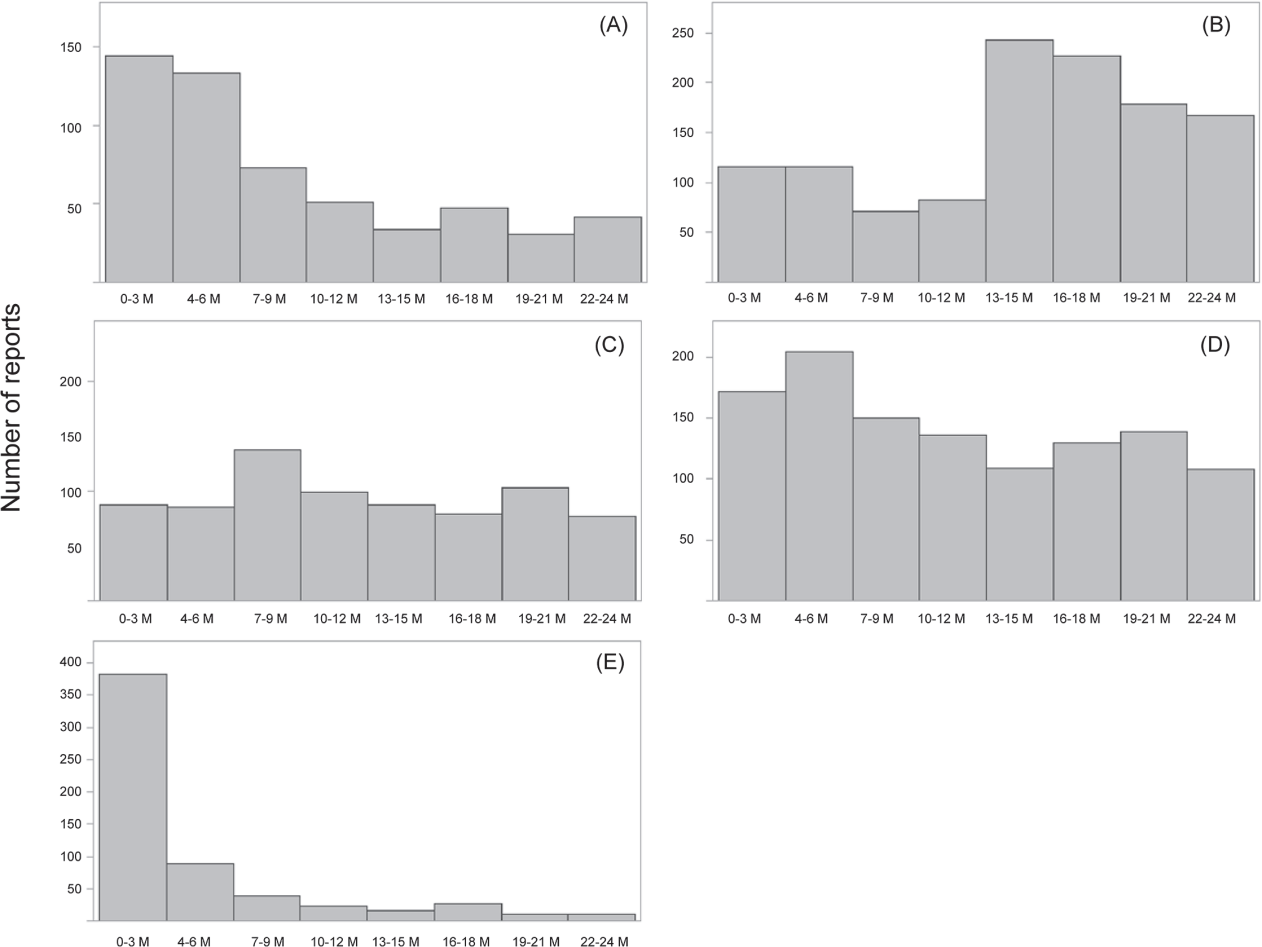
Adverse drug reaction reporting trend for 5 drugs. Total number of individual case safety reports for (A) capecitabine, (B) eldecalcitol, (C) epoetin beta pegol, (D) peginterferon alfa-2a, and (E) sevelamer hydrochloride.

The number of ADR reports for each product are shown in Fig 1. For capecitabine, peginterferon alpha-2a, and sevelamer hydrochloride, the number of ADR reports reached its peak within 6 months after marketing.

**Fig 2:**
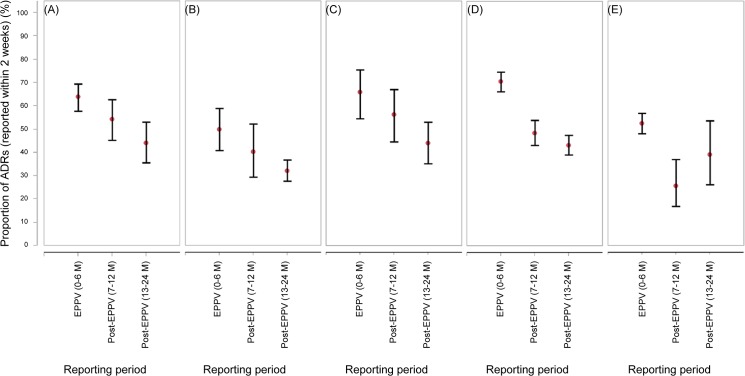
The proportion of adverse drug reactions reported to the manufacturer within 2 weeks of onset. (A) capecitabine, (B) eldecalcitol, (C) epoetin beta pegol, (D) peginterferon alfa-2a, and (E) sevelamer hydrochloride. Error bars represent the 95% confidence intervals.

For all 5 drugs, the proportion of ADRs reported to the manufacturer within 2 weeks of onset was higher in the EPPV period (0–6 months) than in the post-EPPV period (7–12 months) as shown in Fig 2 (for more detail, see S3 Table).

### The effects of ADR seriousness, prominence, and frequency

**Fig 3:**
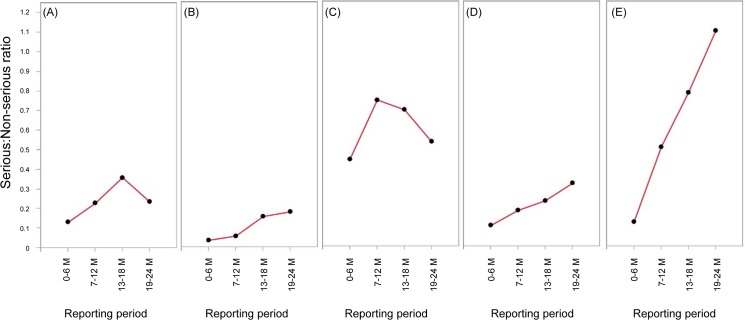
Serious: Non-serious ratios over time. (A) capecitabine, (B) eldecalcitol, (C) epoetin beta pegol, (D) peginterferon alfa-2a, and (E) sevelamer hydrochloride.

For all 5 drugs, we observed an increase in Serious:Non-serious ratio during the post-EPPV period (7–24 months) as shown in Fig 3. The Serious:Non-serious ratio for eldecalcitol, peginterferon alpha-2a, and sevelamer hydrochloride increased steadily throughout the study period. Although there were some decrease for capecitabine and epoetin beta pegol, the decreased values were still higher than those observed in the EPPV period (0–6 month).

**Fig 4:**
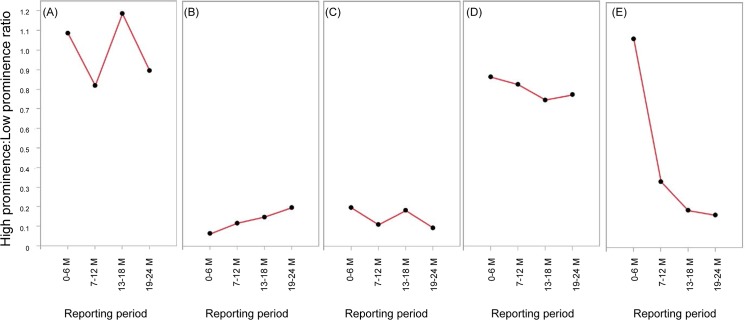
High prominence:Low prominence ratios over time. (A) capecitabine, (B) eldecalcitol, (C) epoetin beta pegol, (D) peginterferon alfa-2a, and (E) sevelamer hydrochloride.

Regarding the High prominence:Low prominence ratio, no clear pattern could be ascribed to the reporting changes (Fig 4). The High prominence:Low prominence ratio for only sevelamer hydrochloride showed a steady decrease during the study period.

**Fig 5:**
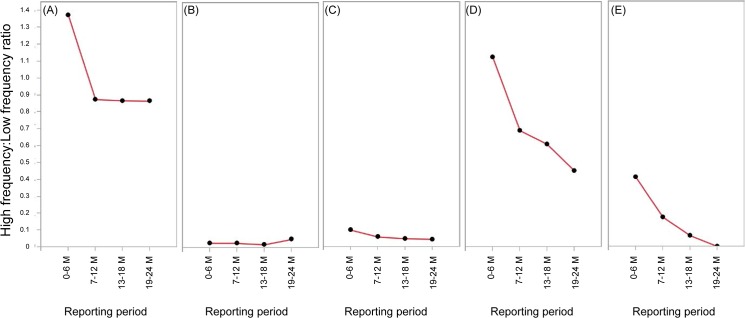
High frequency:Low frequency ratios over time. (A) capecitabine, (B) eldecalcitol, (C) epoetin beta pegol, (D) peginterferon alfa-2a, and (E) sevelamer hydrochloride.

The High frequency:Low frequency ratio for peginterferon alpha-2a and sevelamer hydrochloride decreased steadily throughout the study period (Fig 5).

## Discussion

Although spontaneous reporting databases are a fundamental resource for post-marketing pharmacovigilance, few studies have looked at how reporting trends change over time. Our results suggest several possible explanations of the characteristics seen in spontaneous reporting in Japan.

The number of ADR reports reached its peak within 6 months after marketing for 3 of the 5 drugs studied. EPPV might have the positive effect of shifting this peak earlier than that seen with the typical Weber effect, which shows ADR reporting peak during the 1 to 2 years after marketing [3]. However, this result should be interpreted with caution because we did not have a comparator (i.e., a non-EPPV drug) in this study. During study planning we recognized this would be an issue because most new drugs approved after 2001 were subject to EPPV as required by the Japanese health authority. Therefore, it was impossible to place a comparator in the study. The increase in reporting seen after approximately 13 to 15 months of marketing eldecalcitol was mainly caused by a marked increase in prescriptions after regulatory authorities, on 1 April 2012, lifted a restriction limiting single prescriptions to a 2-week supply. The sevelamer hydrochloride package insert was updated 4 months after initial marketing to include “intestinal perforation” in the Important Precautions and Clinically Significant Adverse Reactions sections, and this could have increased ADR reporting during the EPPV period.

The proportion of ADRs reported to the manufacturer within 2 weeks of onset was higher in the EPPV period (0–6 months) than in the post-EPPV period (7–12 months). Although we could not isolate the EPPV effect from the effects of the early marketing stage, these results may reflect a possible correlation with EPPV.

The Serious:Non-serious ratios increased in the post-EPPV period. This finding was consistent with a previous study that analyzed a French ADR database [5] and suggests that healthcare professionals in Japan may also have a tendency to report serious events more than non-serious events.

The High prominence: Low prominence ratios showed no clear patterns throughout the study period for all drugs except sevelamer hydrochrolide. This finding seems to differ from a previous study stating that well-known ADRs are less likely to be reported [15]. We speculate that one potential reason for this difference is the way ADRs are collected and reported in Japan. The majority of spontaneous reports to the Japanese health authority are submitted by pharmaceutical manufacturers, and many fewer reports are reported directly by healthcare professionals. Japanese MRs, who work for the manufacturers, play a central role in ADR reporting because they collect most reports [20]. These MRs give particular attention to prominent ADRs because one of their roles is to facilitate the proper use of drugs by sharing safety information with healthcare professionals. MRs may proactively ask healthcare professionals about the occurrence of prominent ADRs, which could lessen underreporting of such ADRs. However, because our findings on the effect of prominence differ from those in previous research, further analysis is necessary to elucidate any effects.

The High frequency:Low frequency ratios seemed to decrease over time. This was consistent with previous findings that common events are less likely to be reported than uncommon events [2]. However, because the group of high-frequency events comprised a relatively small sample, our findings showed only a small change over time.

Reporting bias could lead to a false positive or masking problem in disproportionality analysis and detection of increased frequency of reporting. We believe that considering the factors studied here is vital to interpretation of such analysis results. Our results support the hypothesis that serious ADRs tend to be reported more than non-serious ADRs over time. Pharmacovigilance specialists should keep this in mind to strengthen their background knowledge and expectations of routine pharmacovigilance.

This study has some limitations. First, the study focused on the single manufacturer’s data, which limits the generalizability of the study results. However, this also has the advantage of producing consistent analysis results for each product, because potential influential factors such as the number and attitudes of MRs and quality of EPPV implementation would be expected to be similar within a single manufacturer. Second, some observed changes over time would result from not only the reporting bias but also true difference in the incidences of ADRs. We did not obtain true incidences of ADRs because information on the denominator was lacking, an inherent limitation of spontaneous reporting data. It is difficult to exclude possible effects from different patient characteristics in the EPPV period and post-EPPV period. In particular, we should consider the possibility of channeling bias in which high risk patients tend to aggregate in the early phase of post-marketing rather than the later phase [21]. This channeling bias could exist in our study and might affect changes in reporting over time caused by actual differences in ADR occurrences. However, we think it is reasonable to assume the presence of reporting bias because if there was no reporting bias, the proportion of serious events reported in the early stages would have been higher. Our reasoning is that the channeling bias mentioned above could have resulted in a group at higher risk for ADRs, and more severe cases, in the early marketing phase. It is also possible that as healthcare professionals gain experience with a drug after it is marketed, they become more accustomed to its proper use, which could decrease the risk of serious events. As a third limitation, the different time periods might affect the reporting patterns because the healthcare professionals’ attitudes could possibly be affected by external factors such as changes in regulations, media attention, or educational interventions. Indeed, the number of ADRs reported to the Japanese health authority has been increasing recently [22], and this might partially reflect an increased awareness of spontaneous ADR reporting among healthcare professionals. Lastly, we also note that in some respects, there is overlap in the concepts of “commonness,” “prominence,” and “frequency” studied here.

In conclusion, factors associated with ADR reporting attitude in Japan might be different from those observed in other countries, and pharmacovigilance specialists should therefore be cautious when comparing data between different countries. It would be beneficial to validate these findings in a prospective study, and further research is warranted to understand factors related to spontaneous reporting of ADRs and potential reporting biases.

## Supporting Information

S1 Fig Changes in volume shipped after marketing for 5 drugs. (A) capecitabine, (B) eldecalcitol, (C) epoetin beta pegol, (D) peginterferon alfa-2a, and (E) sevelamer hydrochloride. The reporting periods in this figure were close but not identical to those in Table 1 and Figs 1 to 5 because the data were only available as total volume shipped between pre-determined cut-off periods (starting points are as follows: (A) capecitabine, 30 Apr 2003; (B) eldecalcitol, 21 Jan 2011; (C) epoetin beta pegol, 20 Jul 2011; (D) peginterferon alfa-2a, 5 Jul 2003; and (E) sevelamer hydrochloride, 1 May 2003). For capecitabine and sevelamer hydrochloride, the data for 19–24 months were not available and were substituted by half the volume shipped in the corresponding 19–30 months, which data were available. On the horizontal axis, grams (g), kilograms (kg).
(TIF)

S1 Table List of high-prominence events. (DOCX)

S2 Table List of high-frequency events. (DOCX)

S3 Table Details on the proportion of adverse drug reactions reported within 2 weeks of onset. (DOCX)
