# Supplementary material for: Bias in Spontaneous Reporting of Adverse Drug Reactions in Japan
Source: PLoS One. 2015 May 1;10(5):e0126413. doi: 10.1371/journal.pone.0126413 (PMC4416713; doi:10.1371/journal.pone.0126413)
Supplement: S1 Table — (DOCX) [file pone.0126413.s002.docx]

**S1 Table. List of high-prominence events**

| Drug | Term in package insert | Corresponding MedDRA SMQ/PT code | Corresponding SMQ/PT term |
| --- | --- | --- | --- |
| Capecitabine | Bone-marrow suppression | 20000023 | Agranulocytosis (SMQ) |
| Capecitabine | Bone-marrow suppression | 20000027 | Haematopoietic cytopenias (SMQ) |
| Capecitabine | Cardiac disorders | 20000049 | Cardiac arrhythmias (SMQ) |
| Capecitabine | Cardiac disorders | 20000004 | Cardiac failure (SMQ) |
| Capecitabine | Cardiac disorders | 20000150 | Cardiomyopathy (SMQ) |
| Capecitabine | Cardiac disorders | 20000043 | Ischaemic heart disease (SMQ) |
| Capecitabine | Cardiac disorders | 20000068 | Torsade de pointes, shock-associated conditions (SMQ) |
| Capecitabine | Cardiac disorders | 20000001 | Torsade de pointes/QT prolongation (SMQ) |
| Capecitabine | Hand-and-foot syndrome | 10033553 | Palmar-plantar erythrodysaesthesia syndrome (PT) |
| Capecitabine | Hemostasis test abnormal, bleeding | 20000038 | Haemorrhages (SMQ) |
| Capecitabine | Hepatic disorders | 20000006 | Drug related hepatic disorders - comprehensive search (SMQ) |
| Capecitabine | Jaundice | 20000009 | Cholestasis and jaundice of hepatic origin (SMQ) |
| Capecitabine | Renal failure | 20000003 | Acute renal failure (SMQ) |
| Capecitabine | Stomatitis | 20000109 | Oropharyngeal disorders (SMQ) |
| Capecitabine | Symptom of dehydration | 10012735 | Diarrhoea (PT) |
| Capecitabine | Symptom of dehydration | 10012174 | Dehydration (PT) |
| Eldecalcitol | Acute renal failure | 20000003 | Acute renal failure (SMQ) |
| Eldecalcitol | Calculus urinary | 10007027 | Calculus urinary (PT) |
| Eldecalcitol | Hypercalcaemia | 10020583 | Hypercalcaemia (PT) |
| Epoetin beta pegol | Anaphylactic-like reaction | 20000021 | Anaphylactic reaction (SMQ) |
| Epoetin beta pegol | Aplasia pure red cell | 10002965 | Aplasia pure red cell (PT) |
| Epoetin beta pegol | Blood pressure increased | 20000147 | Hypertension (SMQ) |
| Epoetin beta pegol | Brain hemorrhage | 20000064 | Haemorrhagic cerebrovascular conditions (SMQ) |
| Epoetin beta pegol | Hypertensive encephalopathy | 10020803 | Hypertensive encephalopathy (PT) |
| Epoetin beta pegol | Myocardial infarction | 20000047 | Myocardial infarction (SMQ) |
| Epoetin beta pegol | Shock | 20000071 | Anaphylactic/anaphylactoid shock conditions (SMQ) |
| Epoetin beta pegol | Shunt occlusion | 10040621 | Shunt occlusion (PT) |
| Epoetin beta pegol | Thrombosis in dialysis device | 10062546 | Thrombosis in device (PT) |
| Peginterferon alfa-2a | Acute renal failure | 20000003 | Acute renal failure (SMQ) |
| Peginterferon alfa-2a | Agranulocytosis | 20000023 | Agranulocytosis (SMQ) |
| Peginterferon alfa-2a | Agranulocytosis | 20000027 | Haematopoietic cytopenias (SMQ) |
| Peginterferon alfa-2a | Angina pectoris | 20000043 | Ischaemic heart disease (SMQ) |
| Peginterferon alfa-2a | Autoimmune phenomena | 10018916 | Haemolytic anaemia (PT) |
| Peginterferon alfa-2a | Autoimmune phenomena | 10074667 | Immune thrombocytopenic purpura (PT) |
| Peginterferon alfa-2a | Autoimmune phenomena | 10009900 | Colitis ulcerative (PT) |
| Peginterferon alfa-2a | Autoimmune phenomena | 10039073 | Rheumatoid arthritis (PT) |
| Peginterferon alfa-2a | Autoimmune phenomena | 10042945 | Systemic lupus erythematosus (PT) |
| Peginterferon alfa-2a | Autoimmune phenomena | 10047115 | Vasculitis (PT) |
| Peginterferon alfa-2a | Brain haemorrhage | 20000061 | Central nervous system haemorrhages and cerebrovascular conditions (SMQ) |
| Peginterferon alfa-2a | Cardiac arrhythmias | 20000049 | Cardiac arrhythmias (SMQ) |
| Peginterferon alfa-2a | Cardiac failure | 20000004 | Cardiac failure (SMQ) |
| Peginterferon alfa-2a | Cardiomyopathy | 20000150 | Cardiomyopathy (SMQ) |
| Peginterferon alfa-2a | Cerebral infarction | 20000063 | Ischaemic cerebrovascular conditions (SMQ) |
| Peginterferon alfa-2a | Coma | 10010071 | Coma (PT) |
| Peginterferon alfa-2a | Confusion | 10010305 | Confusional state (PT) |
| Peginterferon alfa-2a | Convulsions | 20000079 | Convulsions (SMQ) |
| Peginterferon alfa-2a | Convulsions | 20000212 | Generalised convulsive seizures following immunisation (SMQ) |
| Peginterferon alfa-2a | Delirium | 20000133 | Noninfectious encephalopathy/delirium (SMQ) |
| Peginterferon alfa-2a | Demented symptom | 10057668 | Cognitive disorder (PT) |
| Peginterferon alfa-2a | Depression | 20000167 | Depression (excl suicide and self injury) (SMQ) |
| Peginterferon alfa-2a | Diabetes mellitus | 20000041 | Hyperglycaemia/new onset diabetes mellitus (SMQ) |
| Peginterferon alfa-2a | Disorientation | 10013395 | Disorientation (PT) |
| Peginterferon alfa-2a | Disturbance of consciousness | 10001854 | Altered state of consciousness (PT) |
| Peginterferon alfa-2a | Dyspnoea | 10013968 | Dyspnoea (PT) |
| Peginterferon alfa-2a | Endocarditis | 10014665 | Endocarditis (PT) |
| Peginterferon alfa-2a | Epileptic stroke | 20000079 | Convulsions (SMQ) |
| Peginterferon alfa-2a | Epileptic stroke | 20000212 | Generalised convulsive seizures following immunisation (SMQ) |
| Peginterferon alfa-2a | Gastrointestinal haemorrhage | 20000108 | Gastrointestinal haemorrhage (SMQ) |
| Peginterferon alfa-2a | Hallucination | 20000117 | Psychosis and psychotic disorders (SMQ) |
| Peginterferon alfa-2a | Hematochezia | 20000108 | Gastrointestinal haemorrhage (SMQ) |
| Peginterferon alfa-2a | Hepatic disorders | 20000006 | Drug related hepatic disorders - comprehensive search (SMQ) |
| Peginterferon alfa-2a | Hepatitis | 10019717 | Hepatitis (PT) |
| Peginterferon alfa-2a | Hepatitis aggravated | 20000006 | Drug related hepatic disorders - comprehensive search (SMQ) |
| Peginterferon alfa-2a | Infiltration of the lung | 20000042 | Interstitial lung disease (SMQ) |
| Peginterferon alfa-2a | Interstitial lung disease | 20000042 | Interstitial lung disease (SMQ) |
| Peginterferon alfa-2a | Ischaemic colitis | 20000144 | Ischaemic colitis (SMQ) |
| Peginterferon alfa-2a | Ischaemic heart disease | 20000043 | Ischaemic heart disease (SMQ) |
| Peginterferon alfa-2a | Melena | 20000108 | Gastrointestinal haemorrhage (SMQ) |
| Peginterferon alfa-2a | Nephrotic syndrome | 10029164 | Nephrotic syndrome (PT) |
| Peginterferon alfa-2a | Pancytopenia | 20000023 | Agranulocytosis (SMQ) |
| Peginterferon alfa-2a | Pancytopenia | 20000027 | Haematopoietic cytopenias (SMQ) |
| Peginterferon alfa-2a | Peptic ulcer | 20000106 | Gastrointestinal ulceration (SMQ) |
| Peginterferon alfa-2a | Platelet decreased | 20000023 | Agranulocytosis (SMQ) |
| Peginterferon alfa-2a | Platelet decreased | 20000027 | Haematopoietic cytopenias (SMQ) |
| Peginterferon alfa-2a | Psoriasis | 10037153 | Psoriasis (PT) |
| Peginterferon alfa-2a | Pulmonary embolism | 20000081 | Embolic and thrombotic events (SMQ) |
| Peginterferon alfa-2a | Retinopathy | 20000158 | Retinal disorders (SMQ) |
| Peginterferon alfa-2a | Sepsis | 10040047 | Sepsis (PT) |
| Peginterferon alfa-2a | Shock | 20000021 | Anaphylactic reaction (SMQ) |
| Peginterferon alfa-2a | Shock | 20000071 | Anaphylactic/anaphylactoid shock conditions (SMQ) |
| Peginterferon alfa-2a | Suicidal ideation | 20000167 | Depression (excl suicide and self injury) (SMQ) |
| Peginterferon alfa-2a | Suicide attempt | 20000167 | Depression (excl suicide and self injury) (SMQ) |
| Peginterferon alfa-2a | Thyroid function abnormal | 10043730 | Thyroid function test abnormal (PT) |
| Peginterferon alfa-2a | Ventricular tachycardia | 20000049 | Cardiac arrhythmias (SMQ) |
| Peginterferon alfa-2a | Ventricular tachycardia | 20000001 | Torsade de pointes/QT prolongation (SMQ) |
| Peginterferon alfa-2a | White blood cell decreased | 20000023 | Agranulocytosis (SMQ) |
| Peginterferon alfa-2a | White blood cell decreased | 20000027 | Haematopoietic cytopenias (SMQ) |
| Sevelamer hydrochloride | Gastrointestinal disorder | 10017944 | Gastrointestinal disorder (PT) |
| Sevelamer hydrochloride | Gastrointestinal disorder | 10010774 | Constipation (PT) |
| Sevelamer hydrochloride | Gastrointestinal disorder | 10000081 | Abdominal pain (PT) |
| Sevelamer hydrochloride | Gastrointestinal disorder | 10000060 | Abdominal distension (PT) |
| Sevelamer hydrochloride | Hyperchloraemic acidosis | 10000486 | Acidosis (PT) |
| Sevelamer hydrochloride | Hyperchloraemic acidosis | 10027417 | Metabolic acidosis (PT) |
| Sevelamer hydrochloride | Hypocalcaemia | 10020947 | Hypocalcaemia (PT) |
