# Supplementary material for: Bias in Spontaneous Reporting of Adverse Drug Reactions in Japan
Source: PLoS One. 2015 May 1;10(5):e0126413. doi: 10.1371/journal.pone.0126413 (PMC4416713; doi:10.1371/journal.pone.0126413)
Supplement: S2 Table — (DOCX) [file pone.0126413.s003.docx]

**S2 Table. List of high-frequency events**

| Drug | Term in package insert | Corresponding MedDRA SMQ/PT code | Corresponding SMQ/PT term |
| --- | --- | --- | --- |
| Capecitabine | Al-P increased | 10059570 | Blood alkaline phosphatase increased (PT) |
| Capecitabine | ALT (GPT) increased | 10001551 | Alanine aminotransferase increased (PT) |
| Capecitabine | AST (GOT) increased | 10003481 | Aspartate aminotransferase increased (PT) |
| Capecitabine | Blood glucose increased | 10005557 | Blood glucose increased (PT) |
| Capecitabine | Decreased appetite | 10061428 | Decreased appetite (PT) |
| Capecitabine | Dizziness | 10013573 | Dizziness (PT) |
| Capecitabine | Haemoglobin decreased | 10018884 | Haemoglobin decreased (PT) |
| Capecitabine | Hand-and-foot syndrome | 10033553 | Palmar-plantar erythrodysaesthesia syndrome (PT) |
| Capecitabine | Lymphocyte decreased | 20000030 | Haematopoietic leukopenia (SMQ) |
| Capecitabine | Nausea | 10028813 | Nausea (PT) |
| Capecitabine | Pigmentation | 10062080 | Pigmentation disorder (PT) |
| Capecitabine | Platelet decreased | 20000031 | Haematopoietic thrombocytopenia (SMQ) |
| Capecitabine | Pyrexia | 10037660 | Pyrexia (PT) |
| Capecitabine | Red blood cell decreased | 20000029 | Haematopoietic erythropenia (SMQ) |
| Capecitabine | Vomiting | 10047700 | Vomiting (PT) |
| Capecitabine | White blood cell decreased | 20000030 | Haematopoietic leukopenia (SMQ) |
| Eldecalcitol | Blood calcium increased | 10005396 | Blood calcium increased (PT) |
| Eldecalcitol | Urine calcium increased | 10050278 | Urine calcium increased (PT) |
| Epoetin beta pegol | Blood pressure increased | 20000147 | Hypertension (SMQ) |
| Epoetin beta pegol | Eosinophil count increased | 10014945 | Eosinophil count increased (PT) |
| Epoetin beta pegol | Shunt occlusion, stenosis | 10040621 | Shunt occlusion (PT) |
| Epoetin beta pegol | Shunt occlusion, stenosis | 10059053 | Shunt stenosis (PT) |
| Peginterferon alfa-2a | Abdominal pain | 10000081 | Abdominal pain (PT) |
| Peginterferon alfa-2a | Abdominal pain upper | 10000087 | Abdominal pain upper (PT) |
| Peginterferon alfa-2a | Administration site reaction | 10001315 | Administration site reaction (PT) |
| Peginterferon alfa-2a | Administration site reaction | 10003041 | Application site erythema (PT) |
| Peginterferon alfa-2a | Alopecia | 10001760 | Alopecia (PT) |
| Peginterferon alfa-2a | ALT (GPT) increased | 10001551 | Alanine aminotransferase increased (PT) |
| Peginterferon alfa-2a | Arthralgia | 10003239 | Arthralgia (PT) |
| Peginterferon alfa-2a | AST (GOT) increased | 10003481 | Aspartate aminotransferase increased (PT) |
| Peginterferon alfa-2a | Back pain | 10003988 | Back pain (PT) |
| Peginterferon alfa-2a | Constipation | 10010774 | Constipation (PT) |
| Peginterferon alfa-2a | Cough | 10011224 | Cough (PT) |
| Peginterferon alfa-2a | Decreased appetite | 10061428 | Decreased appetite (PT) |
| Peginterferon alfa-2a | Dermatitis | 10012431 | Dermatitis (PT) |
| Peginterferon alfa-2a | Diarrhoea, Faeces soft | 10012735 | Diarrhoea (PT) |
| Peginterferon alfa-2a | Diarrhoea, Faeces soft | 10074859 | Faeces soft (PT) |
| Peginterferon alfa-2a | Dizziness | 10013573 | Dizziness (PT) |
| Peginterferon alfa-2a | Electrolyte abnormality (calcium, phosphorus, etc.) | 10005393 | Blood calcium abnormal (PT) |
| Peginterferon alfa-2a | Electrolyte abnormality (calcium, phosphorus, etc.) | 10054823 | Blood phosphorus abnormal (PT) |
| Peginterferon alfa-2a | Gamma-GTP increased | 10017693 | Gamma-glutamyltransferase increased (PT) |
| Peginterferon alfa-2a | Haematocrit decreased | 10018838 | Haematocrit decreased (PT) |
| Peginterferon alfa-2a | Haemoglobin decreased | 10018884 | Haemoglobin decreased (PT) |
| Peginterferon alfa-2a | Headache | 10019194 | Head discomfort (PT) |
| Peginterferon alfa-2a | Hypoaesthesia | 10020937 | Hypoaesthesia (PT) |
| Peginterferon alfa-2a | Infection (bacreria, fulgi, virus, etc.) induction or aggravation | 10021789 | Infection (PT) |
| Peginterferon alfa-2a | Lymphocyte decreased | 20000030 | Haematopoietic leukopenia (SMQ) |
| Peginterferon alfa-2a | Malaise | 10025482 | Malaise (PT) |
| Peginterferon alfa-2a | Myalgia | 10028411 | Myalgia (PT) |
| Peginterferon alfa-2a | Nausea | 10028813 | Nausea (PT) |
| Peginterferon alfa-2a | Neutrophil decreased | 20000030 | Haematopoietic leukopenia (SMQ) |
| Peginterferon alfa-2a | Oropharyngeal pain | 10068319 | Oropharyngeal pain (PT) |
| Peginterferon alfa-2a | Oropharyngeal pain, rhinalgia | 10068319 | Oropharyngeal pain (PT) |
| Peginterferon alfa-2a | Oropharyngeal pain, rhinalgia | 10051496 | Rhinalgia (PT) |
| Peginterferon alfa-2a | Pain | 10033371 | Pain (PT) |
| Peginterferon alfa-2a | Platelet decreased | 20000031 | Haematopoietic thrombocytopenia (SMQ) |
| Peginterferon alfa-2a | Pruritus | 10037087 | Pruritus (PT) |
| Peginterferon alfa-2a | Pyrexia | 10020741 | Hyperpyrexia (PT) |
| Peginterferon alfa-2a | Red blood cell count decreased | 20000029 | Haematopoietic erythropenia (SMQ) |
| Peginterferon alfa-2a | Rhinorrhoea | 10039101 | Rhinorrhoea (PT) |
| Peginterferon alfa-2a | Sleep disorder (insomnia, somnolentia, etc.) | 10040984 | Sleep disorder (PT) |
| Peginterferon alfa-2a | Triglycerides increased | 10005839 | Blood triglycerides increased (PT) |
| Peginterferon alfa-2a | Vomiting | 10047700 | Vomiting (PT) |
| Peginterferon alfa-2a | White blood cell decreased | 20000030 | Haematopoietic leukopenia (SMQ) |
| Sevelamer hydrochloride | Abdominal distension | 10000060 | Abdominal distension (PT) |
| Sevelamer hydrochloride | Abdominal pain | 10000081 | Abdominal pain (PT) |
| Sevelamer hydrochloride | Coprostasis, coprostasis aggravated | 10050248 | Faecal volume decreased (PT) |
| Sevelamer hydrochloride | Diarrhoea, Faeces soft | 10012735 | Diarrhoea (PT) |
| Sevelamer hydrochloride | Diarrhoea, Faeces soft | 10074859 | Faeces soft (PT) |
| Sevelamer hydrochloride | Dyspepsia | 10013946 | Dyspepsia (PT) |
| Sevelamer hydrochloride | Nausea | 10028813 | Nausea (PT) |
