# Supplementary material for: Bias in Spontaneous Reporting of Adverse Drug Reactions in Japan
Source: PLoS One. 2015 May 1;10(5):e0126413. doi: 10.1371/journal.pone.0126413 (PMC4416713; doi:10.1371/journal.pone.0126413)
Supplement: S3 Table — (DOCX) [file pone.0126413.s004.docx]

**S3 Table. Details on the proportion of adverse drug reactions reported within 2 weeks of onset**

| Drug name | Reporting Period | Number of ADRs reported within 2 weeks | Proportion (%) | 95% CI LL (%) | 95% CI UL (%) |
| --- | --- | --- | --- | --- | --- |
| Capecitabine | EPPV(0 - 6M) | 167 | 63.7 | 57.8 | 69.3 |
| Capecitabine | Post-EPPV(7 - 12M) | 65 | 54.2 | 45.3 | 62.8 |
| Capecitabine | Post-EPPV(>12M) | 53 | 44.2 | 35.6 | 53.1 |
| Eldecalcitol | EPPV(0 - 6M) | 57 | 50.0 | 41.0 | 59.0 |
| Eldecalcitol | Post-EPPV(7 - 12M) | 27 | 40.3 | 29.4 | 52.3 |
| Eldecalcitol | Post-EPPV(>12M) | 130 | 32.0 | 27.7 | 36.7 |
| Epoetin beta pegol | EPPV(0 - 6M) | 50 | 65.8 | 54.6 | 75.5 |
| Epoetin beta pegol | Post-EPPV(7 - 12M) | 41 | 56.2 | 44.8 | 67.0 |
| Epoetin beta pegol | Post-EPPV(>12M) | 52 | 44.1 | 35.4 | 53.1 |
| Peginterferon alfa-2a | EPPV(0 - 6M) | 309 | 70.5 | 66.1 | 74.6 |
| Peginterferon alfa-2a | Post-EPPV(7 - 12M) | 156 | 48.4 | 43.0 | 53.9 |
| Peginterferon alfa-2a | Post-EPPV(>12M) | 219 | 43.3 | 39.0 | 47.6 |
| Sevelamer hydrochloride | EPPV(0 - 6M) | 253 | 52.5 | 48.0 | 56.9 |
| Sevelamer hydrochloride | Post-EPPV(7 - 12M) | 18 | 25.7 | 16.9 | 37.0 |
| Sevelamer hydrochloride | Post-EPPV(>12M) | 18 | 39.1 | 26.4 | 53.5 |

*ADR: adverse drug reaction, EPPV: early post-marketing phase vigilance, 95% CI LL: 95% confidence interval lower limit, 95% CI UL: 95% confidence interval upper limit
